# Comparison on the Effectiveness of Intravenous Magnesium Sulphate and Intravenous Dexmedetomidine in Reducing Emergence Agitation in Adults Undergoing Otorhinolaryngology Surgery

**DOI:** 10.1155/anrp/5531396

**Published:** 2026-07-20

**Authors:** Girhanif Amri Yunda, Riyadh Firdaus, Aino Nindya Auerkari

**Affiliations:** ^1^ Department of Anesthesiology and Intensive Care, Dr. Cipto Mangunkusumo Hospital, Universitas Indonesia, Jakarta Pusat, Indonesia, ui.ac.id; ^2^ Division of Neuroanesthesia, Department of Anesthesiology and Intensive Care, Dr. Cipto Mangunkusumo Hospital, Universitas Indonesia, Jakarta Pusat, Indonesia, ui.ac.id; ^3^ Division of Intensive Care, Department of Anesthesiology and Intensive Care, Dr. Cipto Mangunkusumo Hospital, Universitas Indonesia, Jakarta Pusat, Indonesia, ui.ac.id

**Keywords:** adult, dexmedetomidine, emergence agitation, magnesium sulphate, otorhinolaryngology

## Abstract

**Background:**

Emergence agitation (EA) is a phenomenon commonly occurring in otorhinolaryngology surgery. It may increase the risk of injury and prolong recovery. Dexmedetomidine is the standard preventive agent, but limited due to its high cost, limited accessibility, extubation time, and incidence of bradycardia and hypotension. Magnesium sulphate (MgSO_4_) is considered a potential alternative but is there is insufficient research available. This study aimed to compare the effectiveness of intravenous MgSO_4_ and dexmedetomidine in reducing EA.

**Methods:**

This study is a multicentered, double‐blinded, randomized controlled trial involving 64 adult patients undergoing otorhinolaryngology surgery. They were divided into two groups in a 1:1 ratio, with one group receiving MgSO_4_ 20 mg/kgBW/h and another receiving dexmedetomidine 0.5 μg/kgBW/h from induction until the procedure ends. We measured the proportion of EA, onset, and duration of EA, intraoperative ephedrine dose, perioperative heart rate and mean arterial pressure (MAP), pain scores, perioperative fentanyl use, intraoperative sevoflurane use, and extubation time.

**Results:**

The proportion of EA was not significantly different between the MgSO_4_ and dexmedetomidine groups (12.5% vs. 15.6%, *p* > 0.05). No differences in the severity, onset and duration of EA. The reduction of MAP and heart rate were greater in the dexmedetomidine group. The intraoperative ephedrine was significantly lower in the MgSO_4_ group (0 vs. 5 mg, *p* < 0.05). No significant differences in fentanyl and sevoflurane use, postoperative pain scores, and extubation time.

**Conclusion:**

Intravenous MgSO_4_ and dexmedetomidine showed no statistically significant difference in the incidence of EA in adult patients undergoing otorhinolaryngologic surgery.

**Trial Registration:** ClinicalTrials.gov: NCT07544173

## 1. Background

Emergence agitation (EA) involves a state of restlessness, disorientation, and purposeless movements in the early recovery phase from general anesthesia (GA). In the adult population, its incidence ranges from 21.3% to 45.9% globally. EA may cause significant clinical consequences, such as injury to patient or medical staff, accidental medical equipment dislodge, respiratory depression, and increased healthcare costs [1, 2]. Otorhinolaryngology surgeries carry a high risk of EA. A study by Yu in China reported an incidence of 55.4% in adult patients, primarily due to endotracheal tube use and nasal packing, which can cause a choking sensation. Other risk factors include obesity, sevoflurane anesthesia, smoking, advanced age, and a history of psychological disorders [3, 4].

EA typically begins 2–5 min after the cessation of inhaled anesthetic agents and can last for 30–45 min postextubation [4, 5]. Currently, there is no specific diagnostic test to confirm EA in adult patients. Most studies use the Riker Sedation‐Agitation Scale (RSAS), the Richmond Agitation‐Sedation Scale (RASS), or the 4‐point Aono scale to assess it [6–8]. At our teaching hospital, Rumah Sakit dr. Cipto Mangunkusumo (RSCM), RASS is the standard tool for evaluating agitation and delirium. A study by Suhandoko and Maskoen in Indonesia also found that the RASS demonstrated better reliability and validity compared to the Ramsay scale [9].

Dexmedetomidine is a highly selective *α*2 agonist. Various studies have shown that different doses of dexmedetomidine are consistently effective in reducing the incidence of EA [10, 11]. It is superior to propofol in preventing agitation and has a lower risk of respiratory depression compared to fentanyl, making it the preferred standard for managing EA [12–14]. However, its high cost, limited availability, risk of hypotension and bradycardia, and prolonged extubation time are notable limitations [10, 15].

Another drug that can be administered to reduce EA incidence is MgSO_4_. It enhances postoperative analgesia, provides sedation, reduces postoperative agitation, and facilitates better recovery after GA [1, 16, 17]. In addition, MgSO_4_ is more cost‐effective compared to dexmedetomidine and is more widely available across our country, Indonesia.

Direct comparative evidence between magnesium sulphate and dexmedetomidine for the prevention of EA in adults remains limited. Most previous comparative studies were conducted in nonotorhinolaryngologic surgical populations. To the best of our knowledge, no multicenter randomized controlled trial from Southeast Asia has directly compared continuous intraoperative magnesium sulphate and dexmedetomidine infusions for the prevention of EA in adult patients undergoing otorhinolaryngologic surgery. The present study therefore addresses an important regional and clinical evidence gap. In addition, given the lower cost and wider availability of magnesium sulphate, establishing whether it provides comparable effectiveness to dexmedetomidine may have important implications for perioperative practice in resource‐limited settings.

## 2. Methods

This study is an experimental double‐blinded, randomized controlled trial. Participants were consecutively selected from adult patients undergoing elective otorhinolaryngology surgery at the central operating theatre of Cipto Mangunkusumo Hospital (RSCM) and Universitas Indonesia Hospital (RSUI) from May to August 2024. Based on previous literature and an anticipated difference in EA incidence, with a significance level of 5% and power of 80%, a minimum of 58 participants was required. To account for potential dropouts, 10% was added, resulting in a total sample size of 64 participants. Sampling was conducted after receiving approval from the Health Research Ethics Committee of RSCM‐Faculty of Medicine Universitas Indonesia and the RSUI Research Ethics Committee. This trial was prospectively registered at ClinicalTrials.gov.

Patients scheduled for elective otorhinolaryngology surgery were assessed for eligibility. Inclusion criteria included adults aged 18–70 years, ASA physical status of 1 or 2, undergoing GA, and willing to participate in the research. Exclusion criteria were pregnant patients, severe cognitive impairment, allergies to study drugs, drug contraindications (atrioventricular block, sinoatrial node dysfunction, and renal failure), neuromuscular disorders, regular use of beta‐blockers or clonidine, not extubated in operating room (OR) nor postanesthesia care unit (PACU) after procedure, and BMI ≥ 40.

Subjects who met the inclusion and exclusion criteria signed the informed consent, and their information, including psychological history and smoking habits, was documented. They were then prepared for otorhinolaryngology surgery under GA. Randomization was performed using computer‐generated block randomization (block size of four) in a 1:1 ratio for drug allocation: MgSO_4_ (20 mg/kgBW/h) or dexmedetomidine (0.5 μg/kgBW/h). Allocation concealment was ensured using sequentially numbered, opaque, sealed envelopes prepared by an independent investigator not involved in patient care or data collection. The pharmacy team prepared the drugs according to the envelope instructions and labelled them with a research code, ensuring the researchers remained unaware of the drug identities. Both drugs were diluted in normal saline to make a total volume of 40 mL in identical syringes. With a similar infusion mL/h rate, ensuring a double‐blind study design. Data were collected by the researchers during the procedure, while the anesthesia team monitored the patients throughout the surgery.

An intravenous line was established before entering the OR. In the OR, standard monitoring equipment, including blood pressure, ECG, oxygen saturation, etCO2, and temperature devices, was set up. Mean arterial pressure (MAP), heart rate (HR), and sevoflurane consumption were recorded. The study drug infusion (MgSO_4_ 20 mg/kgBW/h or dexmedetomidine 0.5 μg/kgBW/h) was initiated during the preoxygenation phase, prior to induction of anesthesia, and continued throughout the maintenance phase until the end of surgery. Therefore, both agents were administered as a continuous intraoperative infusion spanning induction and maintenance phases, ensuring consistent plasma levels during the emergence period. Dosages were calculated based on total body weight (TBW). Anesthesia was induced with a bolus of fentanyl 2 mcg/kgBW and propofol 2 mg/kgBW, followed by sevoflurane to deepen the anesthesia. The train of four (TOF) was measured if available. Rocuronium 0.6 mg/kgBW was administered once the patient was unconscious, followed by intubation. After intubation, 5 mg of IV dexamethasone and 1 g of IV paracetamol were administered. Anesthesia maintenance was achieved with sevoflurane at a minimum alveolar concentration (MAC) of 0.6–1.1. Fentanyl 1 mcg/kgBW and rocuronium 0.2 mg/kgBW were given intermittently as needed. If MAP or HR decreased by more than 30% from baseline values, 5 mg of ephedrine was administered. All fentanyl and ephedrine doses were recorded in the case report form.

At the end of the procedure, 30 mg of IV ketorolac and 10 mg of IV metoclopramide were administered. Before extubation, neostigmine and atropine were given when the TOF count was 4 and the TOF ratio was at least 0.4 or when the patient had resumed spontaneous breathing. Once the patient’s spontaneous ventilation was adequate, sevoflurane and the study drug were discontinued, and 80% oxygen at 6 L per minute was delivered. Extubation was performed while the patient was conscious. Extubation time was defined as the time from discontinuation of sevoflurane to eye opening on verbal command, obeying simple commands, achievement of a train‐of‐four (TOF) ratio ≥ 0.9, and removal of the endotracheal tube. The researcher assessed the EA using the RASS, with EA defined when RASS ≥ 2. Severe EA was defined as a RASS score ≥ +3, corresponding to very agitated or combative behavior requiring pharmacological intervention. If RASS was ≥ 3, propofol bolus of 0.5 mg/kgBW would be administered to the patient. The onset and duration of agitation, as well as the propofol dosage, were documented in the study report. EA onset is measured when both the study drug and sevoflurane are ceased until the RASS reaches ≥ 2, while EA duration is measured from when agitation begins until RASS < 2.

After the surgery, patients were transferred to the recovery room or PACU for assessment of postoperative pain using a numeric rating scale (1–10 scale), MAP and HR, and EA. Tendon reflexes and muscle strength were also evaluated. If necessary, calcium gluconate was administered if hyporeflexia or areflexia happened. Patients were discharged from the PACU once their Aldrete score reached ≥ 9.

Data analysis was performed using the Fisher’s exact test due to small expected cell counts. The Kolmogorov–Smirnov test was used to assess the normality of numerical data. Normally distributed data were presented as mean and standard deviation (SD) and analyzed using the independent Student’s *t*‐test. Nonnormally distributed data were reported by using median and minimum–maximum (min–max) ranges, and the Mann–Whitney test was applied. A risk difference with 95% confidence interval was calculated for the primary outcome. A *p* value < 0.05 was considered statistically significant. For repeated measurement data, such as MAP, HR, and sevoflurane MAC, the general estimating equations (GEE) were used.

## 3. Results

**FIGURE 1:**
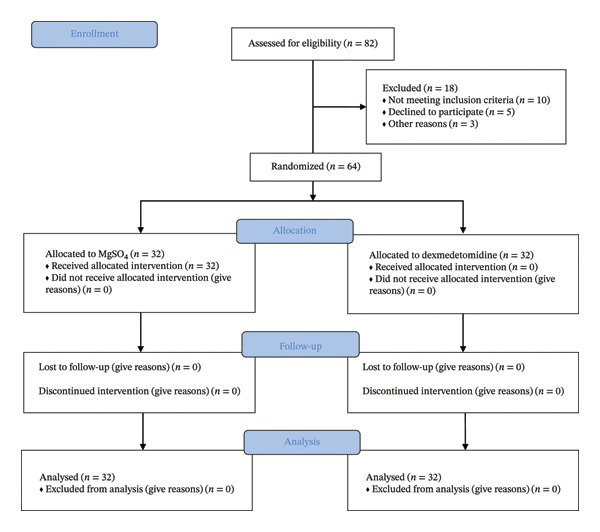
CONSORT 2010 flow diagram.

Data collection was done from May to August 2024 at the Central Operating Theatre of RSCM and RSUI. A total of 82 patients were assessed for eligibility. Eighteen patients were excluded, and 64 patients were randomized into two groups. The participant flow is shown in Figure 1. The baseline demographic and clinical characteristics of the participants are summarized in Table 1.

**TABLE 1:** Subjects characteristics.

Variable	MgSO_4_ (*n* = 32)	Dexmedetomidine (*n* = 32)	*p* value
Age (years)^α^	35 (18–68)	45 (19–65)	0.162
Geriatric (> 60 years old)^β^			1.000
Yes	5 (15.6%)	4 (12.5%)	
No	27 (84.4%)	28 (87.5%)	
Sex^β^			1.000
Male	14 (43.8%)	15 (46.9%)	
Female	18 (56.2%)	17 (53.1%)	
Body weight (Kg)^γ^	61.6 ± 12.7	62.3 ± 14.6	0.842
Height (Cm)^α^	160 (145–181)	165 (141–183)	0.522
Body mass index (BMI)	23.4 ± 4.4	23.4 ± 5.4	0.990
Obesity^β^			1.000
Obese	3 (9.4%)	4 (12.5%)	
Nonobese	29 (90.6%)	28 (87.5%)	
ASA physical status^β^			
ASA 1	10 (31.2%)	9 (28.1%)	1.000
ASA 2	22 (68.8%)	23 (71.9%)	
Surgery duration (min)^α^	210 (50–570)	175 (50–420)	0.336
Smoking status^β^			
Smokers	6 (18.8%)	7 (21.9%)	1.000
Nonsmokers	26 (81.2%)	25 (78.1%)	
Psychologic status^β^			
With psychological disorder	2 (6.3%)	1 (3.1%)	1.000
Without psychological disorder	30 (93.8%)	31 (96.9%)	
Surgery types^β^			0.930
Otology	13 (40.6%)	13 (40.6%)	
Rhinology	14 (43.8%)	15 (46.9%)	
Larynx and pharynx	5 (15.6%)	4 (12.5%)	

^α^Nonnormally distributed data, presented as median (min–max), tested by the Mann–Whitney test.

^β^Data presented as *n* (%), tested by chi‐square test or Fisher exact if cell expectation count < 5.

^γ^Normally distributed data, presented as means ± SD, tested by unpaired student’s *T*‐test.

The occurrence of EA (Table 2) was observed in 12.5% of the MgSO_4_ group, compared to 15.6% of the dexmedetomidine group. The risk difference was −3.1% (95% CI −20.1% to 13.9%). Fisher’s exact test demonstrated no statistically significant difference between groups (*p* = 1.000). In the MgSO_4_ group, 1 subject (3.1%) experienced severe agitation, while 2 subjects (6.2%) in the dexmedetomidine group experienced the same, with no statistically significant difference (*p* = 1.000) (Table 2). Although severe EA occurred in a small number of patients (1 in MgSO_4_ group and 2 in the dexmedetomidine group), the overall median propofol requirement remained 0 mg in both groups, as most patients did not develop agitation requiring pharmacological treatment. The median propofol dose required for all study participants was 0 mg, with no statistically significant difference (*p* = 1.000). There were no statistically significant differences in the onset or duration of EA between the MgSO_4_ and dexmedetomidine groups (*p* = 0.73 and *p* = 0.19, respectively).

**TABLE 2:** EA proportion, severity, propofol use, onset, and duration of EA.

EA proportion^α^					
Group	EA	Without EA	*p*	Risk difference	95% CI
MgSO_4_	4 (12.5%)	28 (87.5%)	1.000	−3.1%	−20.1%–13.9%
Dexmedetomidine	5 (15.6%)	27 (84.4%)			

**Severe EA proportion** **^β^**					

**Group**	**Severe EA**	**Without severe EA**	**p**	**Risk difference**	**95% CI**

MgSO_4_	1 (3.1%)	31 (96.9%)	1.000	−3.2%	−16.8%–10.6%
Dexmedetomidine	2 (6.3%)	30 (93.8%)			

**Propofol use** **^γ^**					

**Variable**	**MgSO**_**4**_ **(*n* = 32)**	**Dexmedetomidine (*n* = 32)**	**p**		

Propofol dose	0 (0–30)	0 (0–30)	1.000		

**Onset and duration of EA** **^δ^**					

**Variable**	**MgSO**_**4**_ **(*n* = 4)**	**Dexmedetomidine (*n* = 5)**	**p**		

Onset (min)	10 (9–13.5)	8 (5–13)	0.73		
Duration (min)	2 (2–3)	5 (1.5–5)	0.19		

^α^Cell expectation count < 5. Analysis was performed using Fisher exact test.

^β^Cell expectation count < 5. Analysis was performed using Fisher exact test.

^γ^Nonnormal data distribution. Analysis was performed using Mann–Whitney test.

^δ^Nonnormal data distribution. Analysis was performed using Mann–Whitney test.

The MAP was measured in mmHg and HR was measured in beats per minute (bpm). Several measurement points were taken, including baseline, preinduction, postinduction, postintubation, postincision, 30 min postincision, postextubation, and 1‐h postoperative. The analysis of the GEE for MAP showed a significant interaction (*p* < 0.005) between dexmedetomidine use and time at postincision, 30 min postincision, and postextubation (Table 3). At postincision, the dexmedetomidine group experienced a 9.4 mmHg decrease in MAP from baseline compared to the MgSO_4_ group (*p* = 0.036). This was also observed at 30 min postincision, with an 8.28 mmHg decrease in the dexmedetomidine group (*p* = 0.032). At postextubation, the dexmedetomidine group showed another reduction of 10.12 mmHg from baseline (*p* = 0/010).

**TABLE 3:** Intraoperative MAP change.

Predictor	Coefficient	Standard error	*p*
Constants	92.78	1.50	< 0.001
Drug			
MgSO_4_	0.0	—	
Dexmedetomidine	−0.281	2.48	0.910
Observation			
Baseline	0.0		
Preinduction	3.43	1.71	0.045
Postinduction	−13.81	2.44	< 0.001
Postintubation	−3.84	2.94	0.192
Postincision	−8.99	3.18	0.005
30 min postincision	−12.40	2.32	< 0.001
Postextubation	0.87	2.94	0.767
1 h postoperative	−5.46	1.88	0.004
Drug and time interactions			
Dexmedetomidine ∗ baseline	0.0		
Dexmedetomidine ∗ preinduction	4.71	2.64	0.075
Dexmedetomidine ∗ postinduction	−2.93	3.62	0.418
Dexmedetomidine ∗ postintubation	−5.68	3.90	0.145
Dexmedetomidine ∗ postincision	−9.40	4.47	0.036^∗^
Dexmedetomidine ∗ 30 min postincision	−8.28	3.85	0.032^∗^
Dexmedetomidine ∗ postextubation	−10.12	3.95	0.010^∗^
Dexmedetomidine ∗ 1 h postoperative	−2.53	2.84	0.374

*Note:* Tested by GEE and exchangeable correlation matrix.

^∗^statistically significant (*p* < 0.05).

The GEE for HR showed a significant interaction between dexmedetomidine use and time at postincision and postextubation (Table 4). At postincision, the dexmedetomidine group had a significant average heart rate reduction of 8.18 bpm from baseline compared to the MgSO_4_ group (*p* = 0.022). This difference reoccurred at postextubation, with an average heart rate drop of 8.84 bpm in the dexmedetomidine group (*p* = 0.008).

**TABLE 4:** Intraoperative HR change.

Predictor	Coefficient	Standard error	*p*
Constants	78.15	1.84	< 0.001
Drug			
MgSO_4_	0.0	—	
Dexmedetomidine	−4.31	2.74	0.910
Observation			
Baseline	0.0		
Preinduction	3.34	2.00	0.095
Postinduction	−2.78	2.00	0.165
Postintubation	8.78	2.56	0.001
Postincision	4.84	2.69	0.072
30 min postincision	−2.50	2.34	0.287
Postextubation	12.75	2.71	< 0.001
1 h postoperative	1.93	1.82	0.287
Drug and time interactions			
Dexmedetomidine ∗ baseline	0.0		
Dexmedetomidine ∗ preinduction	0.15	2.81	0.976
Dexmedetomidine ∗ postinduction	3.75	3.02	0.215
Dexmedetomidine ∗ postintubation	−1.12	3.14	0.742
Dexmedetomidine ∗ postincision	−8.18	3.58	0.022^∗^
Dexmedetomidine ∗ 30 min postincision	0.59	2.97	0.842
Dexmedetomidine ∗ postextubation	−8.84	3.34	0.008^∗^
Dexmedetomidine ∗ 1 h postoperative	−4.06	2.43	0.096

*Note:* Tested by GEE and exchangeable correlation matrix.

^∗^statistically significant (*p* < 0.05).

The most frequent side effects of both study drugs were bradycardia and hypotension (Table 5). When these occurred, 5 mg of ephedrine was given as a rescue agent. The incidence of bradycardia between the dexmedetomidine and MgSO_4_ groups was not statistically significant (*p* = 0.238) according to the Fisher exact test. However, the difference in the proportion of hypotension was significant based on the chi‐square test (*p* = 0.023). Hypotension was observed in 9 cases (28.1%) in the MgSO_4_ group, with a relative risk (RR) 0.474 times lower than in the dexmedetomidine group, which had 19 cases (59.4%). Additionally, dexmedetomidine required a median of 5 mg of ephedrine per surgery, compared to 0 mg for the MgSO_4_ group (*p* = 0.003). No cases of respiratory depression, arrhythmia, or clinically significant hypermagnesemia were observed in either group during the intraoperative or immediate postoperative period.

**TABLE 5:** Proportion of bradycardia and hypotension, intraoperative ephedrine use.

Proportion of bradycardia^α^					
Group	Bradycardia	No bradycardia	*p*	RR	95% CI
MgSO_4_	0 (0%)	32 (100%)	0.238	—	—
Dexmedetomidine	3 (9.4%)	29 (90.6%)			

**Proportion of hypotension** **^β^**					

**Group**	**Hypotension**	**No hypotension**	**p**	**RR**	**95% CI**

MgSO_4_	9 (28.1%)	23 (71.9%)	0.023	0.474	0.254–0.884
Dexmedetomidine	19 (59.4%)	13 (40.6%)			

**Ephedrine intraoperative use** **^γ^**					

**Variable**	**MgSO**_**4**_ **(*n* = 32)**	**Dexmedetomidine (*n* = 32)**	**p**		

Ephedrine dose (mg)	0 (0–30)	5 (0–30)	0.003		

^α^Cell expectation count < 5. Analysis was performed using Fisher exact test.

^β^Cell expectation count ≥ 5. Analysis was performed using chi–square test.

^γ^Nonnormal data distribution. Analysis was performed using Mann–Whitney test.

The use of intraoperative sevoflurane was measured in MAC (Table 6). The sevoflurane MAC is recorded after intubation, during incision, 30 min after the incision, and just before cessation of sevoflurane. The distribution of sevoflurane usage data is not normal. The Mann–Whitney test results indicate no significant difference in MAC between the two groups, and no interaction between MAC and time was found based on the GEE (*p* = 0.075).

**TABLE 6:** Intraoperative sevoflurane use.

MAC	MgSO_4_ (*n* = 32)	Dexmedetomidine (*n* = 32)	*p*
Postintubation	0.8 (0.5–1.1)	0.8 (0.6–1.1)	0.188
During incision	0.9 (0.6–1.1)	0.8 (0.6–1.1)	0.201
30 mins after incision	0.9 (0.6–1.1)	0.8 (0.6–1.1)	0.070
Just before sevoflurane cessation	0.7 (0.4–1.1)	0.6 (0.3–1.1)	0.073
Interaction MAC ∗ time			0.075

*Note:* Nonnormal distribution. Analysis was performed using the Mann–Whitney test. The MAC ∗ time interaction was tested by GEE.

The analysis of fentanyl dosage was based on the intraoperative fentanyl amount in micrograms/kgBW/h and the proportion of patients receiving fentanyl postoperatively as rescue analgesia for severe pain (Table 7). An independent *t*‐test revealed no significant difference in intraoperative fentanyl dosage between the groups (*p* = 0.255). A chi‐square test on postoperative fentanyl use also showed no statistically significant difference between the groups (*p* = 1.000). Postoperative pain scores were assessed using the numeric pain scale, with pain defined as a score of 4 or higher. In both groups, 6.2% of patients reported pain, with no significant difference in proportions (*p* = 1.000).

**TABLE 7:** Intraoperative and postoperative fentanyl use, postoperative pain proportion.

Intraoperative fentanyl use^α^					
Variable	MgSO_4_ (*n* = 32)	Dexmedetomidine (*n* = 32)	*p*		
Intraoperative fentanyl (microgram/kgBW/h)	1.28 ± 0.71	1.09 ± 0.64	0.255		

**Postoperative fentanyl use** **^β^**					

**Group**	**With fentanyl**	**Without fentanyl**	**p**	**RR**	**95% CI**

MgSO_4_	1 (3.1%)	31 (96.9%)	1.000	—	—
Dexmedetomidine	1 (3.1%)	31 (96.9%)			

**Postoperative pain** **^β^**					

**Group**	**Pain**	**No pain**	**p**	**RR**	**95% CI**

MgSO_4_	2 (6.2%)	30 (93.8%)	1.000	—	—
Dexmedetomidine	2 (6.2%)	30 (93.8%)		—	—

^α^Normal data distribution. Analysis was performed using unpaired student’s *t*‐test.

^β^Cell expectation count < 5. Analysis was performed using Fisher exact test.

Extubation time was recorded in minutes, starting from the discontinuation of sevoflurane and the study drug until the patient was fully awake and extubated (Table 8). As the data distribution was nonnormal, the Mann–Whitney test was used for analysis, revealing no significant difference between the two groups (*p* = 0.503).

**TABLE 8:** Extubation time.

Variable	MgSO_4_ (*n* = 32)	Dexmedetomidine (*n* = 32)	*p*
Extubation time (min)	10 (4.5–23)	10 (4–30)	0.503

*Note:* Nonnormal data distribution. Analysis was performed using Mann–Whitney Test.

## 4. Discussion

The overall characteristics of the subjects in both groups were considered comparable, as there were no statistically significant differences (*p* > 0.05). The results showed no significant difference between MgSO_4_ and dexmedetomidine in reducing EA (*p* > 0.05). These findings are consistent with Salman et al., who reported comparable effectiveness between the two agents. In contrast, Ahmed et al. found MgSO_4_ to be superior during percutaneous nephrolithotomy [18]. Variability in surgical type, anesthetic technique, and agitation definitions may explain these differences across studies.

EA has been attributed to asynchronous cortical recovery, where motor and auditory functions return earlier than higher cognitive processing. Dexmedetomidine reduces sympathetic activity through central alpha‐2 receptor stimulation, whereas MgSO_4_ primarily acts as an NMDA receptor antagonist and calcium channel modulator [4, 19]. Despite different mechanisms, both agents may reduce neuronal excitability during anesthetic emergence, which may explain their similar clinical performance.

The overall incidence of EA in this study was relatively low. A meta‐analysis by Shen et al. reported no significant reduction in EA with MgSO_4_; however, only eight studies were included, and postoperative pain was not evaluated [16]. Mahmoud et al. demonstrated dexmedetomidine superiority in pediatric patients, suggesting age‐related differences in neurophysiological response. The low event rate observed in the present study may have limited the ability to detect modest between‐group differences [20].

No significant difference was found in propofol requirements or severe agitation incidence. Similar reductions in EA severity have been reported with dexmedetomidine and MgSO_4_ in previous studies by Kim et al. [7, 21]. Hussein Fahmy Kahla et al. also demonstrated that MgSO_4_ reduced agitation severity and postoperative opioid consumption, indicating combined sedative and analgesic effects [22]. In our cohort, postoperative pain incidence was low, which may have contributed to the overall low agitation rate.

In this study, the dexmedetomidine group demonstrated a more significant reduction in MAP and heart rate compared to the MgSO_4_ group. Based on the GEE analysis, dexmedetomidine produced greater reductions in MAP and heart rate compared with MgSO_4_. Its sympatholytic action via presynaptic alpha‐2 receptor stimulation explains this effect [23–25]. These findings align with previous studies and meta‐analyses demonstrating significant MAP and HR reductions with dexmedetomidine [26–28]. In contrast, MgSO_4_ was administered without a loading dose and at a lower maintenance infusion (< 30 mg/kg), which may explain its milder hemodynamic effect. The greater magnitude of hemodynamic reduction observed with dexmedetomidine is clinically relevant, as it likely contributed to the higher incidence of hypotension and increased requirement for ephedrine in this group [29–31]. Although postoperative hypotension did not differ significantly between groups, dexmedetomidine‐induced hypotension has been reported at higher cumulative doses [15].

Ephedrine was required more frequently in the dexmedetomidine group, likely reflecting stronger sympatholytic activity. Ahmed et al. similarly reported bradycardia requiring vasoactive intervention [18]. However, other studies found no significant difference in hypotension incidence [28, 32]. Overall, MgSO_4_ appeared to provide greater hemodynamic stability, which may be advantageous in patients sensitive to blood pressure fluctuations.

Sevoflurane is known to increase EA risk [2]. In this study, MAC values were comparable between groups. Experimental data suggest that sevoflurane increases neuronal excitability, which may be attenuated by NMDA antagonists or sedative agents [33]. Both MgSO_4_ and dexmedetomidine may reduce anesthetic requirements and contribute to smoother emergence [23, 34]. However, anesthetic depth monitoring was not used, and MAC adjustments were based on hemodynamic parameters, which may introduce variability.

At extubation, MAC values were 0.7 in the MgSO_4_ group and 0.6 in the dexmedetomidine group, with no significant difference observed. High end‐of‐surgery sevoflurane concentrations have been associated with increased agitation and delayed recovery [35, 36]. Although depth‐of‐anesthesia monitoring (e.g., bispectral index or Conox) may optimize volatile agent titration and reduce EA risk, anesthetic depth in this study was guided by hemodynamic parameters rather than EEG‐based monitoring. While MAC values were comparable between groups, variability in individual anesthetic sensitivity may have influenced emergence characteristics and represents a potential confounding factor.

No significant differences were found in intraoperative or postoperative fentanyl consumption. Although dexmedetomidine has demonstrated opioid‐sparing effects in other surgical settings, otorhinolaryngologic procedures generally require lower opioid requirements [23, 37]. Postoperative pain is a recognized risk factor for EA [38, 39]. In this study, postoperative pain occurred in only 6.2% of patients, with postoperative opioid administration in 3.1%, which may partly explain the overall low incidence of agitation.

Median extubation time was 10 min in both groups, with no significant difference. Extubation time was defined as the interval from discontinuation of sevoflurane to eye opening on verbal command, ability to obey simple commands, achievement of TOF ratio ≥ 0.9, and removal of the endotracheal tube. Salman and Mohamed reported longer extubation with dexmedetomidine, whereas other studies have shown no consistent prolongation [1, 40]. The absence of delayed recovery in this study may be related to the continuous low‐dose infusion of dexmedetomidine (0.5 μg/kg/h) without a loading dose [35, 41].

This study provides novel evidence by evaluating magnesium sulphate and dexmedetomidine specifically in adult otorhinolaryngologic surgery, a population at high risk for EA. In addition, the multicenter design enhances the generalizability of the findings. The comparable effectiveness observed between the two agents suggests that magnesium sulphate may serve as a practical and accessible alternative to dexmedetomidine, particularly in resource‐limited healthcare settings.

Several limitations should be acknowledged. The inclusion of different surgical procedures may have introduced heterogeneity in pain intensity and operative duration. The relatively low incidence of EA resulted in a limited number of events, which may have reduced the statistical power to detect moderate between‐group differences. Preoperative anxiety was also not assessed, and intraoperative blood loss, which may have contributed to hypotensive episodes, was not quantified. Additionally, although MAC values were recorded, total sevoflurane consumption and objective anesthetic depth were not measured, leaving residual confounding related to anesthetic depth. Formal assessment of blinding accuracy was not performed; however, identical drug preparation, labeling, and allocation concealment procedures were implemented to minimize the risk of unblinding. Future studies incorporating more homogeneous surgical populations and depth‐of‐anesthesia monitoring may provide more precise estimates of comparative effectiveness.

## 5. Conclusion

This study demonstrated that MgSO_4_ and dexmedetomidine showed comparable effectiveness in reducing EA in adult patients undergoing otorhinolaryngologic surgery. There were no significant differences in the proportion of EA events or the propofol dose required for severe agitation between the two groups. The onset and duration of agitation were also comparable. Patients in the dexmedetomidine group experienced greater decrease in MAP and HR and required higher doses of ephedrine. There were no significant differences in intraoperative use of fentanyl or sevoflurane between the groups. Similarly, postoperative pain and fentanyl use were comparable. The extubation times were also similar, averaging around 10 min for both groups.

## Author Contributions

Riyadh Firdaus: conceptualization, methodology, supervision, investigation, formal analysis, manuscript drafting, and final approval of the manuscript.

Aino Nindya Auerkari: study design, data collection, interpretation of data, manuscript review, and critical revision.

Girhanif Amri Yunda: data collection, statistical analysis, literature review, manuscript editing, and interpretation of data.

## Funding

The authors receive no sponsorship and financial support.

## Disclosure

All authors read and approved the final manuscript.

## Ethics Statement

This study was approved by the Health Research Ethics Committee of Rumah Sakit Universitas Indonesia (RSUI), Indonesia, with ethical approval number S‐064/KETLIT/RSUI/IV/2024 and protocol number 2024‐04‐065, approved on 17 April 2024. Research permission was also granted by Dr. Cipto Mangunkusumo National General Hospital (RSCM), Jakarta, Indonesia, under document number YR.02.01/D.IX.2.3/462/2024. The study was conducted in accordance with the ethical principles of the Declaration of Helsinki and Good Clinical Practice guidelines. Written informed consent was obtained from all participants prior to enrollment in the study.

## Conflicts of Interest

The authors declare no conflicts of interest.

## Data Availability

Research data are not shared.
